# Psychometric evaluation of an item bank for computerized adaptive testing of the EORTC QLQ-C30 cognitive functioning dimension in cancer patients

**DOI:** 10.1007/s11136-017-1648-8

**Published:** 2017-07-13

**Authors:** Linda Dirven, Mogens Groenvold, Martin J. B. Taphoorn, Thierry Conroy, Krzysztof A. Tomaszewski, Teresa Young, Morten Aa. Petersen, on behalf of the EORTC Quality of Life Group

**Affiliations:** 1 Department of Neurology, Leiden University Medical Center, PO BOX 9600, 2300 RC Leiden, The Netherlands; 2 Department of Neurology, Haaglanden Medical Center, The Hague, The Netherlands; 3 Department of Palliative Medicine, Bispebjerg Hospital, Copenhagen, Denmark; 4 Department of Public Health, University of Copenhagen, Copenhagen, Denmark; 5 Medical Oncology Department, Institut de Cancérologie de Lorraine 6, Vandoeuvre-Lès-Nancy Cedex, France; 6 Health Outcomes Research Unit, Department of Gerontology, Geriatrics, and Social Work, Faculty of Education, Ignatianum Academy, Krakow, Poland; 7 Lynda Jackson Macmillan Centre, Mount Vernon Hospital, Northwood, Middlesex UK

**Keywords:** Computerized adaptive testing, Cancer, Cognitive functioning, Item bank, Health-related quality of life, EORTC QLQ-C30

## Abstract

**Background:**

The European Organisation of Research and Treatment of Cancer (EORTC) Quality of Life Group is developing computerized adaptive testing (CAT) versions of all EORTC Quality of Life Questionnaire (QLQ-C30) scales with the aim to enhance measurement precision. Here we present the results on the field-testing and psychometric evaluation of the item bank for cognitive functioning (CF).

**Methods:**

In previous phases (I–III), 44 candidate items were developed measuring CF in cancer patients. In phase IV, these items were psychometrically evaluated in a large sample of international cancer patients. This evaluation included an assessment of dimensionality, fit to the item response theory (IRT) model, differential item functioning (DIF), and measurement properties.

**Results:**

A total of 1030 cancer patients completed the 44 candidate items on CF. Of these, 34 items could be included in a unidimensional IRT model, showing an acceptable fit. Although several items showed DIF, these had a negligible impact on CF estimation. Measurement precision of the item bank was much higher than the two original QLQ-C30 CF items alone, across the whole continuum. Moreover, CAT measurement may on average reduce study sample sizes with about 35–40% compared to the original QLQ-C30 CF scale, without loss of power.

**Conclusion:**

A CF item bank for CAT measurement consisting of 34 items was established, applicable to various cancer patients across countries. This CAT measurement system will facilitate precise and efficient assessment of HRQOL of cancer patients, without loss of comparability of results.

**Electronic supplementary material:**

The online version of this article (doi:10.1007/s11136-017-1648-8) contains supplementary material, which is available to authorized users.

## Introduction

One of the most frequently used tools to measure health-related quality of life (HRQoL) in cancer patients is the European Organisation for Research and Treatment of Cancer Quality of Life Questionnaire (EORTC QLQ-C30) [1]. This questionnaire comprises 30 items organized into five functional scales (physical, role, emotional, cognitive, and social functioning), three symptom scales (fatigue, nausea and vomiting, pain), one overall health/quality-of-life scale, and six single items (dyspnea, insomnia, appetite loss, constipation, diarrhea, and financial difficulties). Traditionally, patients complete all 30 items, allowing comparability of scores across patients. However, limitations of this method are that some patients may have to answer irrelevant questions and that certain domains may be measured with less precision than desired.

These limitations can be overcome with computerized adaptive testing (CAT) [2–4]. CAT is a method to select an individual item set for each patient. Based on the patient’s responses to previous items, the computer program selects a new item from an item bank, aiming to maximize the information obtained. The advantage of CAT is that fewer items are needed to obtain precise measurement and that scores across patients are directly comparable, even if patients do not answer the same subset of items. This is enabled with item response theory (IRT) methods [5].

Currently, the EORTC quality of life group (QLG) is developing CAT versions of all EORTC QLQ-C30 scales, except the overall health/quality-of-life scale [6–15]. To do so, a unidimensional item bank is developed for each scale, consisting of items covering the same aspects of the dimension as reflected by the items of the original scale. To ensure a homogeneous format and compatibility of items with the original QLQ-C30 items, new items are formulated with the same response format and timeframe as the original items.

The EORTC CAT development takes place in an international, cross-cultural setting and consists of four phases: (I) conceptualization and literature search, (II) operationalization, (III) pre-testing, and (IV) field-testing. The first three phases for the development of an item bank for the QLQ-C30 cognitive functioning (CF) scale have been completed [6]. Phase I retrieved 294 items from existing instruments focusing on the subdomains memory and concentration. Following a multistep item selection procedure, most items were excluded, mainly because of redundancy. The steps in phase II included the categorization of items into aspects of cognitive complaints or ‘other’ (step 1), deletion of redundant items (step 2), formulation of items fitting the QLQ-C30 item style (step 3), rating of the items following the continuum of cognitive complaints (step 4), generation of new items in case of insufficient coverage of the measurement continuum (step 5), and lastly expert evaluation, in which items were assessed for their relevance to the construct of cognitive complaints, their appropriateness, completeness, and for whether they were clear and well defined (step 6). In phase III, the preliminary items list was pre-tested in a sample of international cancer patients to determine the appropriateness of the selected items for the target population and to ensure content validity. Based on the remaining items, 43 new items were formulated. These were pre-tested in a group of cancer patients. Phase III resulted in a list of 44 items (including the two original QLQ-C30 CF items) measuring CF in cancer patients. Here we report the results on the phase IV field-testing and psychometric evaluations of these 44 candidate items for the CF item bank, which will be used in CAT measurement.

## Methods

The methods used are in accordance with the general approach used for psychometric analyses of item banks for CAT, as previously reported for other EORTC QLQ-C30 scales [9, 11–14].

### Sample

The EORTC CAT is developed for international use in cancer patients. According to the guidelines, a heterogeneous sample of cancer patients across Europe (Denmark, Poland, France, and the United Kingdom) was included with different diagnoses, stages of disease, treatment modalities, and sociodemographic factors. To be eligible, patients had to be over 18 years with a histologically verified cancer, and were required to be physically and mentally fit enough (no formal screening procedure was used, but patients’ health status was judged by the physician or researcher) to complete the questionnaire. To assure sufficient coverage of patients with different characteristics and to obtain precise calibration of the IRT model, a minimum of 1000 patients were included [16–18]. Local ethics committees of the participating countries approved the study and written informed consent was obtained before participation.

### Questionnaire

Patients were asked to complete a questionnaire consisting of the 42 newly developed items on CF [6], next to the two original CF items, and five debriefing items asking whether patients found any of the items problematic. Twelve out of the 42 items were related to concentration and 30 to memory, and were fitted to the QLQ-C30 item style with a recall period of a week and the use of a 4-point Likert scale ranging from ‘not at all’ to ‘very much.’ In addition, information on patient and disease characteristics was collected.

### Statistical analysis

The psychometric analyses for the selection and calibration of the items for the CF item bank consisted of six steps:

1. Descriptive and basic statistical analyses

Descriptive statistics were used to define the patient population, to calculate response rates and item means and standard deviations (SD), and to determine correlations between the items and the original QLQ-C30 CF sum scale.

2. Evaluation of dimensionality and local dependence

The aim was to find a unidimensional solution including both original QLQ-C30 CF items and as many new items as possible. Dimensionality of the items was assessed using factor analysis methods for ordinal categorical data [19] including exploratory evaluations of dimensionality examining eigenvalues and scree plot [20]. These were followed by confirmatory methods where a reasonable fit of a unidimensional model was defined as follows: the root mean square of approximation (RMSEA) <0.10, the Tucker-Lewis Index (TLI) >0.90 and the comparative fit index (CFI) >0.90 [21, 22]. Since standard IRT models require that items are locally independent (i.e., item responses are independent when controlling for the overall level of CF), we also evaluated the residual correlations from the final factor model. Residual correlations <0.20 were defined as indication of local independence [23].

3. Calibration of the IRT model and evaluation of item fit

Besides local independence, IRT models also assume monotonicity. This is the increasing likelihood for an item response reflecting good CF with increasing CF score. Monotonicity was evaluated by checking the average item score in relation to the ‘rest score,’ i.e., the sum score of all items except the evaluated item. Compliance with monotonicity implies that an average item score should not decrease for increasing values of the rest score [24].

A polytomous IRT model, the generalized partial credit model (GPCM) [25], was used as basis for the CF CAT. In this type of model, each item has a slope parameter to describe the item’s ability to discriminate between subjects with different levels of CF, and a set of threshold parameters which define where on the CF continuum neighbor response options are equally likely to be selected. The average of an item’s threshold is termed the item location.

Parscale (Scientific Software International [SSI], Skokie, IL, USA) was used to estimate the IRT model [18]. Item fit was examined using the item-fit test S-*χ* ^2^ [26] implemented for polytomous items in the SAS macro IRTfit [27]. In addition, bias and indices of fit were evaluated, by calculating the difference between expected and observed item responses and the infit and outfit indices, respectively [28]. Infit and outfit are both statistics based on squared standardized residuals across patients, i.e., they reflect the difference between the model expected responses and the actual observed responses to an item. Although similar, the infit is more sensitive to responses from respondents with CF scores close to the item’s location, while the outfit is more sensitive to unexpected responses far from the item’s location. The infit is therefore particularly important, since it reflects the principle of CAT measurement, where items closest to the respondents actual CF score are asked. Infit and outfit values between 0.7 and 1.3 were defined acceptable. Although smaller values (<0.07) indicate ‘overfit’ (i.e., better fit than expected statistically, because of redundancy), these are not as worrisome as larger values (>1.3), which indicate misfit to the model.

4. Test for differential item functioning

Differential item functioning (DIF), i.e., whether items are perceived and behave similarly in different patient groups, was tested using ordinal logistic regression methods for gender, age, country, cancer site, cancer stage, current treatment, cohabitation, educational level, and work. Each item was entered as the dependent variable and group (DIF) variables as independent variables, controlling for the CF score estimated using the calibrated IRT model in the previous step. DIF was defined potentially relevant if *p* < 0.001 (because of a large sample and multiple testing) *and* if the regression coefficient for the group variable was moderate to large, i.e., *β* > 0.64 (for group variables with more than two categories, at least two categories’ coefficients should differ >0.64) [29, 30]. For each item, each group variable was first tested individually for both uniform and non-uniform DIF. Because confounding of group variables may cause false-positive DIF findings, significant group variables in the individual tests were entered simultaneously in a multivariable logistic regression model. Only the findings of these models are reported.

Moreover, the possible effect of DIF findings on the estimation of CF was evaluated [31]. Although DIF may have significant impact on item level, this may be neglectable on scale level. Therefore, CF scores obtained with the model in step 3 (not accounting for DIF) were compared with scores obtained with a model accounting for DIF. If the CF estimates of these two models differed more than the median standard error of the CF estimates (the median standard error used to represent the general uncertainty of the CF estimates), referred to as ‘salient scale-level differential functioning’ [11, 12, 14, 31], this was regarded as problematic.

5. Evaluation of discarded items

To ensure that items have not been discarded erroneously in the previous steps, the discarded items were added one at the time to the list of items obtained after step 4 in order to evaluate whether the item still showed misfit. If discarded erroneously, items could be included again.

6. Evaluation of measurement properties of the CAT

The information function, a measure of the measurement precision of an item or set of items at different levels of CF, of the final CF item bank was calculated. High measurement precision was defined as an information score >20, corresponding to a reliability of >95% [32].To further evaluate the measurement properties of the final CF CAT, simulations of CAT administration based on the collected data were performed. CATs were simulated with 1 up to 33 items (total of 33 simulations) and then the scores based on these CATs were compared with the score based on all 34 items. Relative validity (RV), the ratio of two test statistics for comparing two groups, of these CATs as compared to the QLQ-C30 CF scale for detecting expected group differences was estimated [33]. When using the *t* test statistic of each CAT as the numerator and the t-test for the QLQ-C30 CF scale as denominator, an RV value >1 indicates that smaller samples may be needed using the CAT measures to obtain the same power as with the QLQ-C30 CF scale. To evaluate the RV of the CATs compared to the QLQ-C30 scale, we compared groups expected to differ (known groups) based on the following hypotheses: patients not on treatment would have better CF than patients on treatment, patients with stage I or II would have better CF than patients with stage III or IV disease, younger patients would have better CF than older patients, patients working would have better CF than patients not working, and patients with more years of education would have better CF than those with less years. Only known group variables that were significant for at least one of the outcomes (QLQ-C30 CF score or one of the CAT-based scores) were used to calculate RVs. In addition to these evaluations based on the observed data, we also evaluated the RV of the CATs based on simulated data across different groups and group sizes [9].

## Results

A total of 1030 cancer patients were included in this study. Detailed description of patient characteristics is presented in Table 1. The results follow the stepwise outline as presented in the Methods section.

**Table 1:** Clinical characteristics of the 1030 participating patients

Characteristic	*N* (%)/mean
Age in years, mean (range)	63 (26–97)
Gender	
Men	488 (47.4%)
Women	542 (52.6%)
Country	
Denmark	138 (13.4%)
France	158 (15.3%)
Poland	280 (27.2%)
United Kingdom	454 (44.1%)
Cancer site	
Breast	237 (23.0%)
Gastrointestinal	144 (14.0%)
Gen-urinary	171 (16.6%)
Gynecological	99 (9.6%)
Hematological	51 (5.0%)
Head and neck	87 (8.4%)
Lung	33 (3.2%)
Other	208 (20.2%)
Cancer stage	
I–II	615 (59.7%)
III–IV	409 (39.7%)
Unknown	6 (0.6%)
Current treatment	
Chemotherapy	378 (36.7%)
Other treatment	337 (32.7%)
No treatment	314 (30.5%)
Unknown	1 (0.1%)
Cohabitation	
Living with a partner	750 (72.8%)
Living alone	267 (25.9%)
Missing	13 (1.3%)
Educational level	
0–10 years	311 (30.2%)
11–13 years	269 (26.1%)
14–16 years	221 (21.5%)
>16 years	225 (21.8%)
Missing	4 (0.4%)
Work	
Fulltime	337 (32.7%)
Part-time	76 (7.4%)
Retired	431 (41.8%)
Other	184 (17.9%)
Missing	2 (0.2%)

1. Descriptive and basic statistical analyses

Response rates for the 44 items ranged from 99.2 to 100%, and item means from 1.1 to 2.0 on a scale from 0 to 3, with higher scores reflecting more cognitive difficulties. In general, patients experienced at most minor cognitive difficulties. Only 7% of the patients reported no problems on any item. All new items correlated >0.4 with the original QLQ-C30 CF scale, and all but two items (item 23, *r* = 0.47; item 43, *r* = 0.56) correlated >0.6. No items were rated as difficult to understand, annoying, confusing, upsetting, or intrusive by more than 4 patients (0.4%). Overall, 97% of the patients found all 44 items unproblematic. Based on these results, no items were deleted in this step.

However, seven items had <10 responses in the ‘very much’ category. To avoid too low numbers in the IRT analyses, the ‘quite a bit’ and ‘very much’ categories were combined and used in further analyses for these items.

2. Evaluation of dimensionality and local dependence

Exploratory factor analysis revealed one clearly dominating factor (eigenvalue = 28.1), explaining 64% of the total variation. However, two additional factors also had eigenvalues >1, explaining 4.7 and 2.4% of the variance, respectively (see also Supplementary Table 1). The scree plot indicated that two factors were required to explain the variation in the data. This was supported by the finding that a one-factor solution showed poor fit indices: RMSEA = 0.104, CFI = 0.872, and TLI = 0.985. Alternatively, a two-factor model seemed to fit well: RMSEA = 0.065, CFI = 0.936, and TLI = 0.994. In this two-factor model, the two original QLQ-C30 CF items load on different factors: one primarily focusing on memory and the other focusing primarily on concentration. As the aim was a unidimensional item bank covering both subdomains (as the original QLQ-C30 CF scale), the selection strategy in the confirmatory factor analysis focused on discarding items having very low loading on one factor in a two-factor solution; items tapping on both subdomains would make the item bank more homogenous while still covering both subdomains. For example, the item ‘Have you had difficulty remembering the names of common things?’ did not seem to involve concentration and was therefore discarded, while the item ‘Have you had difficulty performing two tasks simultaneously, e.g. having a conversation while cooking?’ seemed to cover both concentration and memory and was included. Using this strategy and the predefined criteria for model fit (i.e., RMSEA <0.10, and TLI and CFI >0.90), 34 items could be included in a unidimensional model (RMSEA = 0.095, CFI = 0.903, and TLI = 0.989), explaining 66% of the total variation (eigenvalue = 22.5).

All 561 residual correlations for the 34 items were <0.20 except one which was 0.24. This indicates no or at most trivial local dependence among the retained items.

3. Calibration of the IRT model and evaluation of item fit

Although some items exhibited minor deviations from monotonicity, likely due to random variation, no items were deleted. Therefore, a GPCM was calibrated to the 34 items (details on item fit are summarized in Table 2). The item-fit tests showed that all items had an acceptable fit (*p* > 0.10 for *χ* ^2^ test) and they were therefore retained in the model. Next, bias estimates were all close to zero, indicating no or negligible systematic bias. The infit ranged from 0.91 to 1.15 and the outfit from 0.73 to 1.20. Thus, results indicate acceptable fit for all 34 items.

**Table 2:** Parameter estimates and fit statistics for the 34 items in the final IRT model

Item	Slope	Location	Item fit *p* value	Infit	Outfit
*Item 1* Have you had difficulty performing two tasks simultaneously, e.g. having a conversation while cooking?	1.55	−1.57	0.813	1.01	1.03
*Item 3* Have you been distracted by thoughts when you should have been concentrating on something else?	1.73	−0.99	0.726	0.97	0.92
*Item 5* Have you had difficulty remembering what date it was?	1.59	−1.41	0.640	1.06	0.97
*Item 8* Have you had difficulty remembering what somebody told you a few minutes earlier?	2.01	−1.52	0.980	0.98	0.90
*Item 9* Have you had difficulty remembering what you were going to say while you were talking?	2.25	−1.23	0.392	1.01	0.89
*Item 10* Have you had difficulty remembering what happened the last few days?	1.97	−1.53	0.965	0.99	0.83
*Item 11* Have you walked into a room but forgotten what you went for?	1.78	−0.83	0.609	0.98	0.94
*Item 12* Have you had difficulty remembering the names of relatives, friends, or other people you see regularly?	1.24	−1.56	0.689	0.98	0.90
*Item 13* Have you had difficulty remembering what you initially were doing if you started to do something else in the meantime?	2.25	−1.56	0.714	1.01	0.88
*Item 14* Have you had difficulty remembering what you were doing when you were interrupted?	2.66	−1.36	0.602	0.99	0.80
*Item 15* Have you had difficulty in concentrating on things, like reading a newspaper or watching television? (q20)	1.84	−1.45	0.106	1.05	1.00
*Item 16* Have you been reading something and had to read the same lines again because you were distracted?	1.83	−1.11	0.374	0.96	0.92
*Item 18* Have you had difficulty remembering things? (q25)	2.68	−1.00	0.880	0.97	0.86
*Item 19*: Have you had difficulty maintaining concentration even when something really interested you?	3.15	−0.91	0.588	0.99	0.84
*Item 24* Have you been forgetful?	2.52	−1.12	0.139	1.03	0.96
*Item 25* Have you had difficulty paying attention on a task or a conversation for a longer period of time?	2.48	−1.29	0.718	0.93	0.85
*Item 26* Have you had difficulty recognising relatives, friends, or other people you see regularly?	1.63	−2.40	0.973	1.15	1.20
*Item 27* Have you had difficulty remembering what someone just told you?	3.32	−1.14	0.554	1.07	0.81
*Item 28* Have you had difficulty paying attention for as long as you wanted or needed to?	2.50	−1.36	0.389	1.05	1.02
*Item 30* Have you had difficulty remembering new information, like a person’s name or simple instructions?	2.31	−1.30	0.590	0.97	0.87
*Item 31* Have you had difficulty remembering to take things you needed with you?	1.66	−1.19	0.260	0.99	0.90
*Item 32* Have you become distracted from a task before finishing it?	2.08	−1.55	0.412	0.93	0.84
*Item 33* Have you had difficulty remembering whether you had already done something?	2.48	−1.66	0.998	1	0.86
*Item 34* Have you had difficulty remembering something you had just said?	2.62	−1.30	0.964	0.94	0.73
*Item 35*: Have you had difficulty remembering to pass on a message or remind someone of something?	1.82	−1.55	0.764	0.93	0.85
*Item 36* Have you had difficulty maintaining concentration even when doing something important?	3.74	−1.46	0.837	0.98	0.90
*Item 37* Have you had difficulty remembering what you were just thinking?	2.91	−1.49	0.918	0.94	0.81
*Item 38* Have you had difficulty gathering your thoughts?	2.15	−1.05	0.334	0.91	0.89
*Item 39* Have you had difficulty remembering to do the things you had planned to do?	2.78	−1.47	0.995	0.95	0.79
*Item 40* Have you had difficulty remembering what weekday it was?	1.80	−1.45	0.933	0.96	0.87
*Item 41* Have you had difficulty remembering what a text you were reading was about?	1.88	−1.76	0.686	1.05	0.94
*Item 42* Have you had difficulty remembering what you did a few days earlier?	2.22	−1.56	0.562	1.01	0.94
*Item 43* Have you forgotten to do routine things such as turning off the light or locking the door?	1.20	−1.76	0.926	0.96	0.88
*Item 44* Have you had difficulty staying focused on a task or an activity?	3.01	−1.40	0.363	0.95	0.74

4. Test for differential item functioning

Eighteen items showed significant DIF (all uniform), but only for age, country, or work (Table 3). Most differences were found for country (*n* = 13), followed by age (*n* = 7) and work (*n* = 1). Three items, showing the most pronounced indications of DIF, were evaluated for their possible effect on CF estimation. Results showed that the potential DIF for these items had negligible impact on CF estimation (CF scores accounting for and not accounting for DIF all correlated ≥0.99). Therefore, all items were retained in the model.

**Table 3:** Results of the DIF analysis

Item	DIF	*β*	*p* value	DIF	*β*	*p* value
Item 1	Country	−0.76 (Poland vs. rest)	<0.0001			
Item 3	Age	0.80 (≥70 vs. rest)	<0.0001			
Item 5	No DIF					
Item 8	Country	1.15 (Poland vs. rest)	<0.0001			
Item 9	No DIF					
Item 10	Country	1.33 (Poland vs. rest)	<0.0001			
Item 11	No DIF					
Item 12	Age	−1.26 (<50 vs. ≥50)	<0.0001			
Item 13	No DIF					
Item 14	No DIF					
Item 15 (q20)	Age	0.93 (<70 vs. ≥70)	<0.0001	Country	0.93 (Poland vs. rest)	<0.0001
Item 16	No DIF					
Item 18 (q25)	Age	1.67 (<40 vs. ≥40)	<0.0001			
Item 19	Work	0.69 (Retired vs. rest)	0.0002			
Item 24	Country	−0.74 (Poland vs. rest)	<0.0001			
Item 25	No DIF					
Item 26	No DIF					
Item 27	No DIF					
Item 28	Country	−1.56 (Poland vs. rest)	<0.0001			
Item 30	Age	−1.38 (<40 vs. ≥40)	0.0020	Country	−0.85 (Denmark vs. rest)	<0.0001
Item 31	No DIF					
Item 32	No DIF					
Item 33	No DIF					
Item 34	Country	−0.82 (Poland vs. rest)	<0.0001			
Item 35	No DIF					
Item 36	Country	0.71 (Poland vs. rest)	0.0006			
Item 37	Country	0.95 (Denmark vs. rest)	<0.0001			
Item 38	Age	0.75 (<50 vs. ≥50)	0.0002	Country	2.57 (Poland vs. rest)	<0.0001
Item 39	No DIF					
Item 40	No DIF					
Item 41	Country	1.08 (Denmark & France vs. Poland & United Kingdom)	<0.0001			
Item 42	Country	0.78 (Denmark & France vs. Poland & United Kingdom)	<0.0001			
Item 43	No DIF					
Item 44	Age	1.49 (<40 vs. ≥40)	0.0002			

One beta for each group variable (e.g., country) is presented which summarizes the potential DIF, as well as the reference category that was used in each case

5. Evaluation of discarded items

Adding any of the ten discarded items to the model again, resulted in significantly poorer model fit and lack of unidimensionality. Therefore, no items were restored, and the 34 items (Table 2) comprise the final item bank. The item bank includes 11 items on concentration and 23 items on memory.

6. Evaluation of measurement properties

**Fig. 1:**
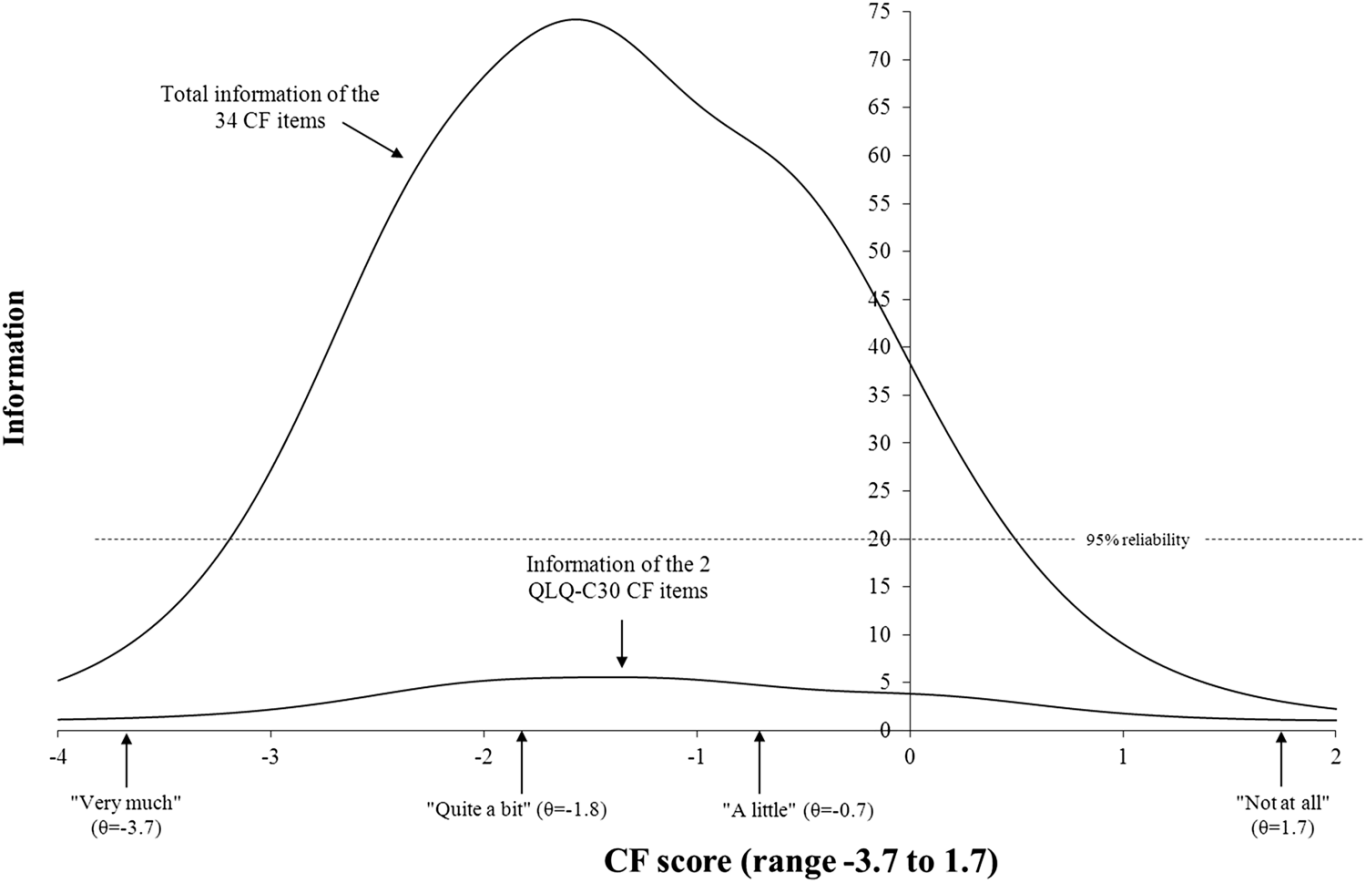
Test information function for the 34 items in the final model and information on the two original QLQ-C30 cognitive functioning (CF) items. CF scores for all response options (ranging from ‘not at all’ to ‘very much’) are presented and their level of measurement precision

In Fig. 1, the test information function for the 34 items in the final model is displayed as well as the information function on the two original QLQ-C30 CF items. CF scores ranged from −3.7 (‘very much’ on all items) to 1.7 (‘not at all’). The total test scale has very high measurement precision for scores from −3.2 to 0.5 (about 3.7 standard deviation units). This means that the item bank is particularly precise for patients with at least minor cognitive difficulties, and less precise for patients with very few cognitive difficulties. The item bank results in markedly higher measurement precision than the two original QLQ-C30 CF items across the whole continuum.

**Fig. 2:**
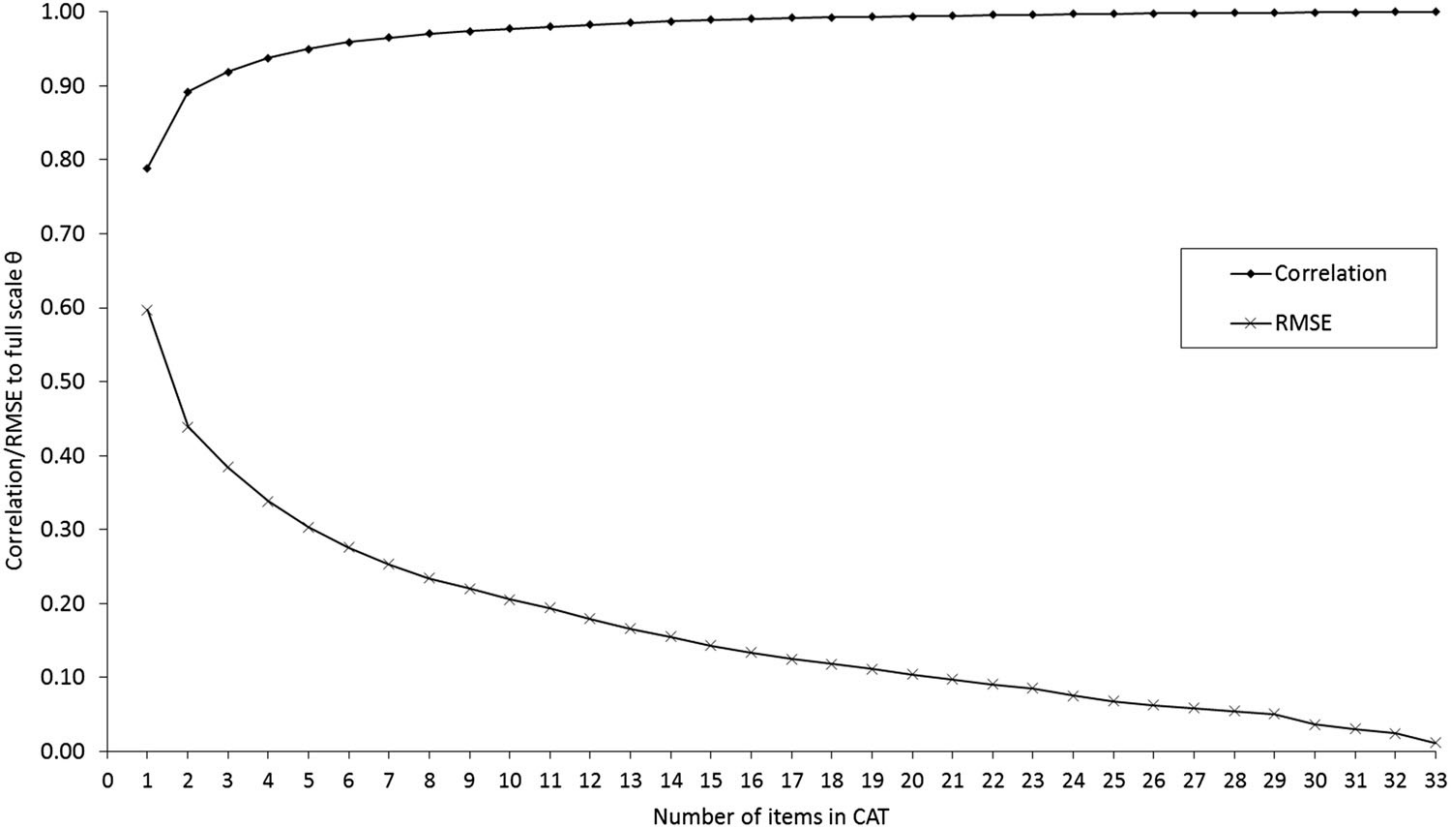
Correlations and root mean square errors (RMSEs) of *θ*’s based on fixed-length CATs and the cognitive functioning score based on all 34 items. For example, scores based on three or more items correlated highly (>0.90) with the score based on all items

**Fig. 3:**
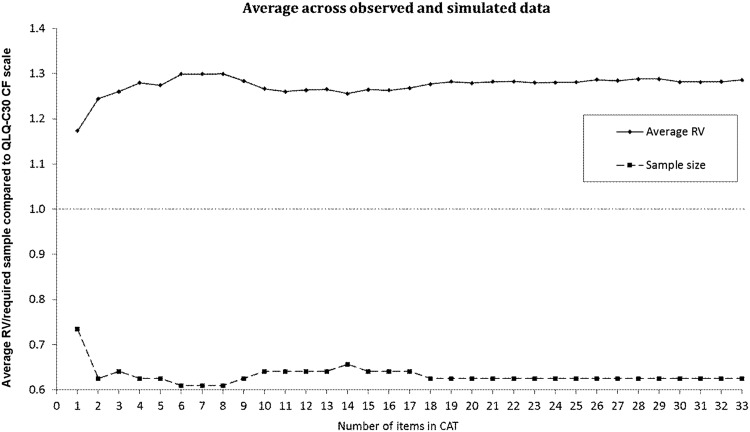
The average relative validity (RV) and relative required sample size using CAT measurement across observed and simulated data, compared to using the QLQ-C30 cognitive functioning sum scale. For example, using a CAT with two items, the data show that the validity of CAT is 1.24 times that of the QLQ-C30 cognitive functioning sum scale (RV = 1.24). Moreover, the required sample size is 37% (sample size = 0.63) smaller using this two-item CAT when compared to the QLQ-C30 cognitive functioning sum scale, while obtaining the same power

The CAT simulations based on collected data showed that scores based on three or more items correlated highly (>0.90) with the score based on all items (Fig. 2). Average RV scores across known groups for the observed and simulated data are shown in Supplementary Figs. 1 and 2, respectively. Average RV scores across known groups for the observed and simulated data combined, across all evaluated settings (different group sizes and group differences), are shown in Fig. 3. For the observed data our hypothesized known group differences were confirmed except that we did not observe any differences in CF between working and not working patients. Across the remaining known groups, the average estimated savings in the sample size without loss of power, based on the observed data, was close to 50% for CATs of all lengths (Supplementary Fig. 1). Simulated data showed savings in the sample size up to about 25% compared to the QLQ-C30 scale (Supplementary Fig. 2). These were very consistent across simulated sample sizes and there were only minor variations across known groups. Although estimated savings varied across methods (≈50 vs. ≈25%), simulations on both observed and simulated data indicated clear reductions in sample size requirements when using CAT to measure CF. On average across methods, the savings were about 35–40% when asking two or more items (Fig. 3).

## Discussion

The overall aim of the EORTC CAT project is to develop item banks for all EORTC QLQ-C30 scales, which can be used for CAT. In this study, we report the psychometric evaluation of 44 candidate items for the CF item bank, which were developed in previous phases [6]. The factor analysis indicated that the candidate items are divided into two subdimensions: memory and concentration. However, 34 of the 44 items sufficiently covered both subdimensions to be included into a unidimensional model. All 34 items had an acceptable fit to the IRT model. Although several items showed DIF, this DIF had negligible impact on CF estimation. Thus, CF scores based on the item bank can be compared across studies, irrespective of patient characteristics.

The measurement precision of the CF item bank was high for patients reporting at least minor cognitive difficulties, and somewhat less precise for patients reporting trivial cognitive difficulties. The majority (73%) of the cancer patients in this study experienced at most minor cognitive difficulties (here defined as a CF score <−0.7, corresponding to answering ‘a little’ to all 34 items in the final item bank). This suggests that the measurement precision for general cancer patients may be suboptimal. The results did show that CAT measurement will be very precise in the subset of cancer patients with some cognitive impairment. This means that CAT could be particularly useful in patients with primary brain tumors and patients with systemic cancer with central nervous system metastases or treatment-related cognitive deficits [34–41]. However, primary brain tumor patients were not included in the patient sample and it is unknown how many patients had brain metastases or treatment-related cognitive deficits, limiting generalizability of the results (i.e., whether the results are also applicable to brain tumor patients). From a methodological point of view, new items that are relevant for patients with trivial cognitive difficulties could be constructed and added to the item bank in order to enhance measurement precision. However, from a clinical point of view this may be irrelevant, because a very low level of cognitive difficulties may not be different from ‘normality,’ and there are no treatment implications for patients with no or minor cognitive difficulties. Overall, the measurement precision of the item bank was much higher than the two original QLQ-C30 CF items alone, across the whole continuum, although this may partially be explained by including items that are similar in content.

Of the 34 items, 11 items focus on concentration and 23 items on memory. This imbalance was not caused by the exclusion of items in the validation process, as only two items on concentration and eight on memory were discarded. To guarantee content balance, CAT may be programmed to systematically select items from both subdomains. A simple solution to ensure direct coverage of both subdomains would be to start a CAT by asking the two original QLQ-C30 CF items. To fit the content covered by the two QLQ-C30 CF items, this item bank also narrows its coverage to concentration and memory, while cognitive functioning comprises more domains [42]. On the other hand, limiting the coverage to the original domains will allow direct comparability with other studies that used the QLQ-C30 to assess HRQoL.

Evaluations of known groups on observed data indicated that using CAT resulted in large savings in study sample sizes, around 50% for CATs of all lengths, as compared to the original QLQ-C30 CF scale, without loss of power. When the sample size would be further reduced, this would be at the expense of statistical power. Simulated data showed somewhat smaller savings, of up to 25%. Although estimated savings varied between the observed and simulated data, sample sizes will already be significantly reduced, on average by 35–40%, when asking at least two items (corresponding to 6% of the CF item bank). Thus, fewer patients would need to be included in studies with cognitive complaints as the primary endpoint. Moreover, response burden for patients may be reduced, as a 1-item CAT can result in a better estimation of CF than the original 2-item QLQ-C30 CF sum scale.

In conclusion, we have developed a CF item bank for CAT measurement consisting of 34 items, applicable to patients with various cancer diagnoses, across different countries. The item bank showed good psychometric properties. Moreover, by tailoring the item set to the individual patients, measurement precision is enhanced and the response burden possibly reduced. When CAT versions for all QLQ-C30 scales are developed, resulting in a complete EORTC QLQ-C30 CAT instrument, these remain to be validated in an independent dataset. Currently, the EORTC Quality of Life Group has initiated such a large validation study.

## Electronic supplementary material

Below is the link to the electronic supplementary material.

Supplementary material 1 (DOCX 96 kb)

Supplementary material 2 (DOCX 101 kb)

Supplementary material 3 (DOCX 11 kb)
