# Supplementary material for: Psychometric evaluation of an item bank for computerized adaptive testing of the EORTC QLQ-C30 cognitive functioning dimension in cancer patients
Source: Qual Life Res. 2017 Jul 13;26(11):2919–29. doi: 10.1007/s11136-017-1648-8 (PMC5655578; doi:10.1007/s11136-017-1648-8)
Supplement: Supplementary file 1 — Supplementary material 1 (DOCX 96 kb) [file 11136_2017_1648_MOESM1_ESM.docx]

**Supplementary Figure 1. The ratio between the average relative validity (RV) and relative required sample size using CAT measurement compared to using the QLQ-C30 cognitive functioning sum scale based on the observed data.**


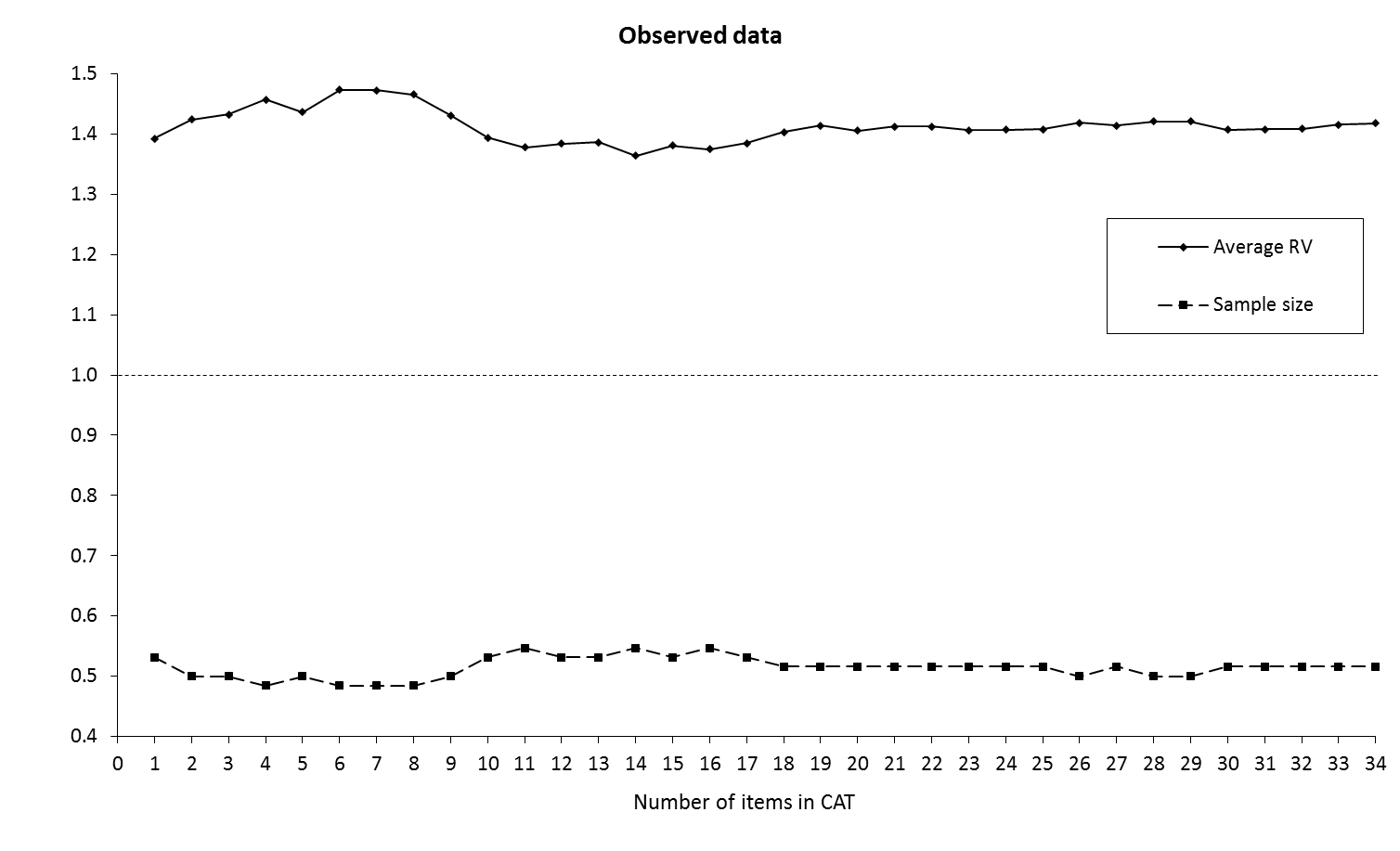


**Average RV/required sample compared to QLQ-C30 CF scale**
