# Supplementary material for: Psychometric evaluation of an item bank for computerized adaptive testing of the EORTC QLQ-C30 cognitive functioning dimension in cancer patients
Source: Qual Life Res. 2017 Jul 13;26(11):2919–29. doi: 10.1007/s11136-017-1648-8 (PMC5655578; doi:10.1007/s11136-017-1648-8)
Supplement: Supplementary file 3 — Supplementary material 3 (DOCX 11 kb) [file 11136_2017_1648_MOESM3_ESM.docx]

| **Factor** | f1 | f2 | f3 | f4 | f5 |
| --- | --- | --- | --- | --- | --- |
| **Eigenvalues** | 28.138 | 2.06 | 1.075 | 0.964 | 0.845 |
| **Difference in eigenvalues** | 26.08 | 0.99 | 0.11 | 0.12 | 0.04 |
| **Eigenvalue ratio f_i-1_/f_i_** | 13.66 | 1.92 | 1.12 | 1.14 | 1.05 |
| **% explained** | 64.0% | 4.7% | 2.4% | 2.2% | 1.9% |
| **Cumulative explained** | 64.0% | 68.6% | 71.1% | 73.3% | 75.2% |

Supplementary Table 1. Results of the explanatory factor analysis
